# Supplementary material for: Causal Relationship between Aging and Anorexia Nervosa: A White-Matter-Microstructure-Mediated Mendelian Randomization Analysis
Source: Biomedicines. 2024 Aug 16;12(8):1874. doi: 10.3390/biomedicines12081874 (PMC11351342; doi:10.3390/biomedicines12081874)
Supplement: Supplementary file 1 [file biomedicines-12-01874-s001.zip › Supplementary Figure/Figure S4/Singe SNP plot of MR analysis for TL to ubm-b-1606.pdf]

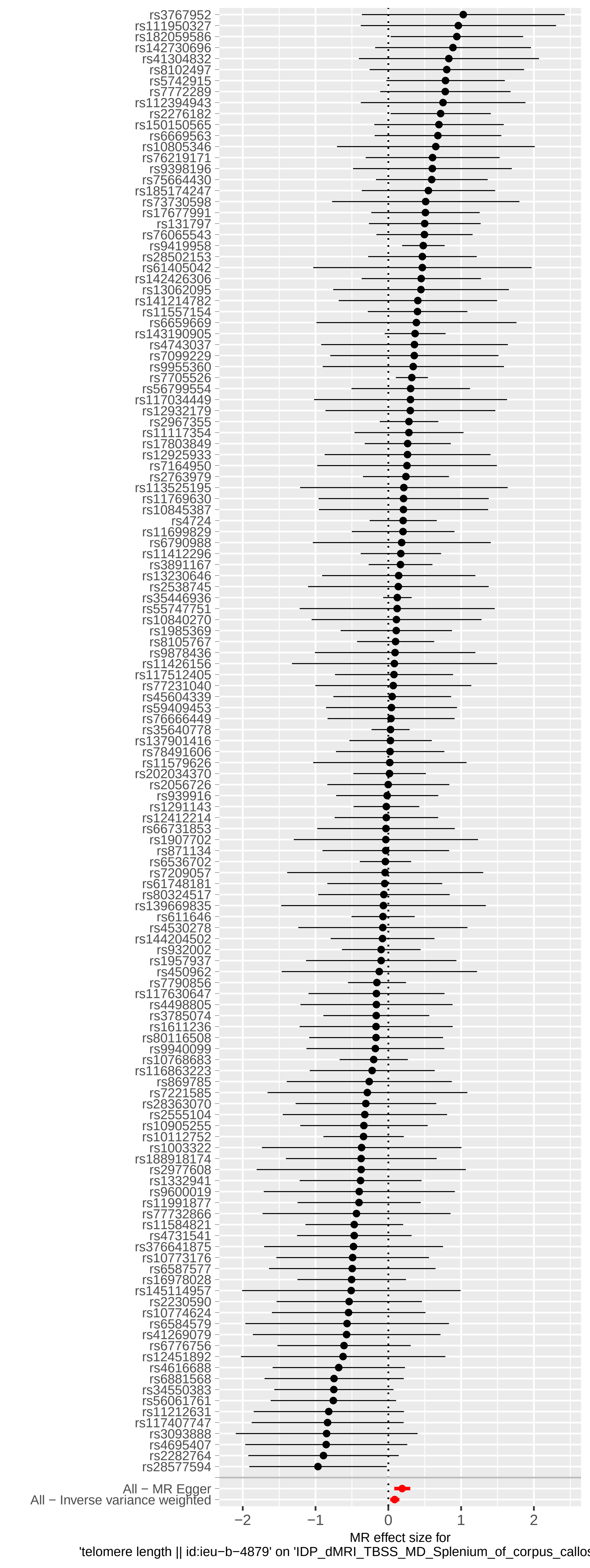
rs3767952
rs111950327
rs182059586
rs142730696
rs41304832
rs8102497
rs5742915
rs7772289
rs112394943
rs2276182
rs150150565
rs6669563
rs10805346
rs76219171
rs9398196
rs75664430
rs185174247
rs73730598
rs17677991
rs131797
rs76065543
rs9419958
rs28502153
rs61405042
rs142426306
rs13062095
rs141214782
rs11557154
rs6659669
rs143190905
rs4743037
rs7099229
rs9955360
rs7705526
rs56799554
rs117034449
rs12932179
rs2967355
rs11117354
rs17803849
rs12925933
rs7164950
rs2763979
rs113525195
rs11769630
rs10845387
rs4724
rs11699829
rs6790988
rs11412296
rs3891167
rs13230646
rs2538745
rs35446936
rs55747751
rs10840270
rs1985369
rs8105767
rs9878436
rs11426156
rs117512405
rs77231040
rs45604339
rs59409453
rs76666449
rs35640778
rs137901416
rs78491606
rs11579626
rs202034370
rs2056726
rs939916
rs1291143
rs12412214
rs66731853
rs1907702
rs871134
rs6536702
rs7209057
rs61748181
rs80324517
rs139669835
rs611646
rs4530278
rs144204502
rs932002
rs1957937
rs450962
rs7790856
rs117630647
rs4498805
rs3785074
rs1611236
rs80116508
rs9940099
rs10768683
rs116863223
rs869785
rs7221585
rs28363070
rs2555104
rs10905255
rs10112752
rs1003322
rs188918174
rs2977608
rs1332941
rs9600019
rs11991877
rs77732866
rs11584821
rs4731541
rs376641875
rs10773176
rs6587577
rs16978028
rs145114957
rs2230590
rs10774624
rs6584579
rs41269079
rs6776756
rs12451892
rs4616688
rs6881568
rs34550383
rs56061761
rs11212631
rs117407747
rs3093888
rs4695407
rs2282764
rs28577594
All − MR Egger
All − Inverse variance weighted
−2
−1
0
1
2
MR effect size for
'telomere length || id:ieu−b−4879' on 'IDP_dMRI_TBSS_MD_Splenium_of_corpus_callos
